# Extracellular matrix assembly during chondrogenic differentiation of amniotic fluid- and adipose-derived mesenchymal stromal cells under TGF-β3 induction

**DOI:** 10.1007/s10735-026-10977-y

**Published:** 2026-10-01

**Authors:** Carolina Coli Zuliani, Ingrid Iara Damas, Kleber Cursino de Andrade, Paulo Kharmandayan, Ângela Maria Moraes, Ali Mobasheri, Ibsen Bellini Coimbra

**Affiliations:** 1 Department of Orthopedics, Rheumatology and Traumatology, Universidade Estadual de Campinas (UNICAMP) - School of Medical Sciences, 50, Vital Brazil Street, Campinas, CEP 13083-887 SP Brazil; 2 Department of Gynecology and Obstetrics, Universidade Estadual de Campinas (UNICAMP) - School of Medical Sciences, 101 Alexander Fleming Street, Campinas, CEP 13083-891 SP Brazil; 3 Universidade Estadual de Campinas (UNICAMP) - School of Medical Sciences, 126 Tessália Vieira de Camargo Street, Campinas, CEP 13083-887 SP Brazil; 4 Department of Engineering of Materials and of Bioprocesses, Universidade Estadual de Campinas (UNICAMP) -School of Chemical Engineering, 500 Albert Einstein Avenue, Campinas, CEP 13083-852 SP Brazil; 5 Department of Personalized Medicine, State Research Center for Innovative Medicine, Vilnius, LT-08406 Lithuania; 6 Health Science and Technology Research Unit, University of Oulu, PO Box 5000, Oulu, FI-90014 Finland; 7 Department of Joint Surgery, First Affiliated Hospital of Sun Yat-sen University, Guangzhou, Guangdong Province, China; 8 Faculty of Medicine, University of Liège, Liège, Belgium; 9 Department of Health Sciences, Medicine and Research, Center for Regenerative Medicine, Faculty of Health and Medicine, Krems University of Continuing Education, Krems, Austria; 10 Department of Orthopedic Surgery, Maastricht University Medical Center, Maastricht, Netherlands

**Keywords:** Chondrogenic differentiation, Amniotic fluid mesenchymal stromal cells, Adipose-derived mesenchymal stromal cells, TGF-β3, Extracellular matrix, Chitosan–xanthan gum scaffold

## Abstract

Osteoarthritis is a major degenerative joint disease often triggered by traumatic injuries, yet current treatment options remain limited in restoring cartilage integrity. Mesenchymal stromal cells (MSCs) represent a promising source for chondrogenic regeneration, but their differentiation potential may vary according to tissue of origin. Here, we compared the chondrogenic differentiation capacity of human amniotic fluid–derived MSCs (hAF-MSCs, CD117⁺ selected) and human adipose-derived MSCs (hAD-MSCs) when cultured within porous chitosan–xanthan gum (CX) scaffolds under TGF-β3 stimulation. Both MSC types were expanded, phenotypically characterized, and induced toward chondrogenesis. Robust adhesion and proliferation within CX scaffolds were observed, accompanied by deposition of extracellular matrix enriched in glycosaminoglycans and collagen fibers. Histological staining (hematoxylin–eosin, alcian blue, picrosirius red, and Masson’s trichrome) and immunohistochemistry confirmed the synthesis of cartilage-specific macromolecules, including collagen type II and aggrecan, while immunofluorescence highlighted a strong collagen II signal. Scanning electron microscopy further revealed extensive cell–matrix interactions and abundant fibrillar organization. These findings demonstrate that both hAF-MSCs and hAD-MSCs undergo effective chondrogenic differentiation within CX scaffolds under TGF-β3 stimulation. Notably, hAF-MSCs exhibited a superior integration and an enhanced extracellular matrix assembly compared to hAD-MSCs, providing new biochemical insights into their extracellular matrix assembly and supporting their potential application in cartilage tissue engineering.

**Supplementary Information:**

The online version contains supplementary material available at https://doi.org/10.1007/s10735-026-10977-y.

## Introduction

Joint injuries are increasingly common in active individuals and have become a major focus of public health concern in recent times because they affect a large portion of the world’s population.(Katz et al. 2021) Joint injuries following trauma represent an important risk factor for the development of arthritic diseases such as osteoarthritis (OA) (Callahan et al. 2019; Hunter et al. 2002; Musumeci et al. 2015). Articular cartilage is a highly specialised connective tissue that lines the ends of long bones in load-bearing joints (Becerra et al. 2010). It is designed for absorbing compressive loads and sheer forces associated with physical activity and joint loading, but it has a poor capacity for self-repair and regeneration, especially in older adults. The degeneration and loss of articular cartilage is a key component of the clinical syndrome of OA, which is one of the most common causes of pain and disability in middle-aged and older people (Buckwalter 1998; Buckwalter et al. 2000, 2005; Katz et al. 2021).OA is characterized by changes in the metabolism of chondrocytes within articular cartilage (Zheng et al. 2021), increasing the expression of proteolytic enzymes and the degradation of extracellular matrix (ECM) macromolecules, resulting in the loss of cartilage tissue (Martin and Buckwalter 2002).

Although OA disease affects all joints tissues and compartments, it is the articular cartilage component which has received most of the attention, especially in the context of its role in providing low friction joint articulation and in how its degradation leads to loss of joint function (Buckwalter et al. 2000). There is rising interest in the concept of joint preservation (Madry et al. 2011), regeneration and remodeling (Luria and Chu 2014) through stimulation of cartilage repair.

There are currently no effective pharmacological disease-modifying OA drugs (Hunter 2011; Karsdal et al. 2016; Oo et al. 2018). Treatments to slow or prevent both the degradation of cartilage and other joint tissues following joint injuries and the development of joint diseases have not been fully established. Currently recommended treatments are generally based on symptom relief (Wang et al. 2021). However, a number of biological drugs and stromal cell-based therapies are emerging for the treatment of OA (Mobasheri 2013; Mobasheri and Batt 2016; Richardson et al. 2010). In the context of joint injuries and cartilage repair, the use of stromal cells represents a promising prospect for future clinical applications. These cells can be combined with polymer-based scaffolds, which are capable of prevent cell dispersion and loss by increasing the number of cells close to the site of injury, with improved regeneration and immunomodulation potential (Lu et al. 2018). In addition, the scaffolds can also support cell development considering issues related to tissue architecture.

The development and use of scaffolds for stimulation of chondrogenic differentiation of mesenchymal stromal cells requires meeting a large set of requirements of the biomaterial (Richbourg et al. 2019; Westin et al. 2017; Yang et al. 2018), including: biocompatibility; biodegradable or bioresorbable features, with controllable degradation and resorption rates; adequate chemical composition, with suitable surface chemistry for cell attachment, proliferation, and differentiation; capacity to mimic the natural microenvironment of the ECM; stability in culture medium for in vitro studies and in the physiological environment after implantation; suitable porosity and roughness; pores with appropriate architecture regarding shape and distribution, size and interconnectivity; mechanical properties matching those of the tissues at the site of implantation, in addition to easy processability to form a variety of shapes and sizes, capacity to resist to sterilizations procedures and competitive costs (Asadi et al. 2018; Zhang et al. 2019). Several biomaterials have been analysed for this purpose, mainly those composed of polysaccharides, such as agarose, alginate, xanthan gum and particularly, chitosan (Peng et al. 2022; Richbourg et al. 2019; Westin et al. 2017). The combination of chitosan and xanthan gum forms a polyelectrolyte complex that has been widely investigated for tissue engineering applications due to its biocompatibility, structural stability, and ability to support cell adhesion and matrix deposition. In cartilage engineering, this combination may help recapitulate features of the glycosaminoglycan-rich extracellular matrix, providing a porous and mechanically stable microenvironment favorable to chondrogenic differentiation.

Different sources of stromal cells are studied for use in cartilage cell therapy (Loo and Wong, 2021, Zhang, et al., 2016). However, some studies have shown that amniotic fluid stromal cells have ideal characteristics for various applications in cell therapy, due to the ease of obtaining, expanding, isolating, maintaining and differentiating them (De Coppi et al. 2007; de Mara et al. 2013; Mara et al. 2010). It is well known that amniotic fluid is composed by different cell types that can be differentiated into other lineages. Among them, multipotent cells characterized by the expression of the CD117 antigen (c-kit) show significant promise (De Coppi et al. 2007).

Another widely used source of mesenchymal stromal cells is the human adipose tissue ( hAD-MSC), that are also characterized by their high capacity for expansion and differentiation (Si et al. 2019; Zuk 2010). The ability to proliferate and differentiate into various mesenchymal lineages makes hAD-MSC a potentially less invasive substitute for hBM-MSC in cell-based therapeutic applications (Mohamed-Ahmed et al. 2018). Transforming Growth Factor-beta 3 (TGF-β3) is one of the principal biochemical stimuli used to induce chondrogenesis.

Among the transforming growth factor-beta (TGF-β) isoforms, TGF-β3 is considered one of the most potent inducers of chondrogenic differentiation in mesenchymal stromal cells (MSCs), promoting the expression of cartilage-specific genes and extracellular matrix production (Chen et al. 2016; Mackay et al. 1998; Zha et al. 2021). TGF-β3 initiates this process through binding to TGF-β receptors I and II, leading to Smad2/3 phosphorylation and subsequent formation of the Smad2/3–Smad4 complex, which translocated to the nucleus to regulate the expression of key chondrogenic genes, including SOX9, COL2A1 and ACAN (Chen et al. 2016; Gurusinghe and Strappe 2014) In addition to the canonical Smad pathway, signalling networks involving MAPK and other regulatory pathways contribute to chondrogenic commitment and extracellular matrix synthesis (Kondo et al. 2015; Watanabe et al. 2001; Zha et al. 2021). Recent studies have also highlighted the role of TGF-β3 in cartilage homeostasis and osteoarthritis, reinforcing its importance for cartilage tissue engineering. (Du et al. 2023) Moreover, incorporating TGF-β3 into biomaterial-based delivery systems, particularly natural polymer-derived scaffolds, enhances growth factor retention and sustained release, creating a microenvironment that favours stable chondrogenesis and cartilage matrix deposition (Almeida et al. 2014, 2017; Dahlin et al. 2014).

However, to the best of our knowledge, there are no studies comparing the chondrogenic differentiation process of hAF-MSC with that of hAD-MSC. In this study, we assessed the potential of hAF-MSC and hAD-MSC stromal cells for chondrogenic differentiation directly into chitosan-xanthan gum scaffolds designed for cartilage tissue engineering, under TGF-β3 stimulation, a growth factor that is involved in chondrogenesis in vivo and is responsible for maintaining the stability of the ECM that is formed, preventing its calcification and degeneration (Almeida et al. 2014, 2017).

## Materials and methods

### Harvesting, isolation and expansion of human amniotic fluid mesenchymal stromal cells

All studies were conducted in compliance with recognized international standards, including the guidelines of the World Medical Association (WMA) and the principles of the Declaration of Helsinki. For this study (approved by the Ethical Committee of the University of Campinas, São Paulo, Brazil CAAE no.: 31984414.6.0000.5404), hAF-MSC samples were obtained from pregnant women in the second trimester of pregnancy submitted to amniocentesis under formal medical indication from the Centre for Integral Attention to Women’s Health at the University of Campinas (Campinas, SP), after signing the Free and Informed Consent (FIC) form. Samples containing 10 mL of amniotic fluid were submitted to the laboratory, centrifuged to obtain a cell pellet, which was suspended in 5 mL of alpha minimum essential medium (α-MEM) (GIBCO), supplemented with 20% fetal bovine serum (FBS) (GIBCO) and 1% penicillin-streptomycin-glutamine (P/S)(GIBCO) and maintained in an incubator at 37 °C with a humid atmosphere at 5% CO_2_.The cells were removed from the culture flask with 0.25% Trypsin-EDTA (GIBCO) and submitted to immunomagnetic separation (MACS-Miltenyi Biotec^®^) for CD117 (c-kit) according to the manufacturer’s protocol. Cells were expanded up to the fourth passage for characterization and application in chondrogenic differentiation experiments.

### Sourcing, isolation and expansion of human adipose-derived mesenchymal stromal cells

hAD-MSC were obtained from liposuction material of patients at the Clinic Hospital of the University of Campinas, after signing the FIC form. The samples were processed according to the protocol of Zuk et al. (2013), which consists in repeated washes and enzymatic digestion with 0.075% type II collagenase (Sigma-Aldrich, St. Louis, MO). The stromal cells obtained were maintained in the same culture conditions as above with low glucose DMEM (GIBCO) medium, supplemented with 10% FBS (GIBCO) and 1% P/S. Similar to hAF-MSCs, hAD-MSCs were expanded at a 1:3 ratios for three consecutive passages and utilized at the fourth passage (P4) for all experimental procedures to ensure phenotypic stability.

### Characterization of mesenchymal stromal cells

For morphological analysis, adherent cell cultures from both sources were systematically monitored by optical microscopy to maintain 70% confluence.

For differentiation into the three mesenchymal lineages, we used StemPro Adipogenic Differentiation Kit™ (Gibco), StemPro Chondrogenic Differentiation Kit™ (Gibco) and StemPro Osteogenic Differentiation Kit™ (Gibco) commercially available, in accordance with the manufacturer’s instructions. Cell differentiation into adipocytes, chondrocytes and osteocytes was evaluated after 21 days of culture with Oil Red O, Alcian Blue and Alizarin Red staining, respectively.

For immunophenotypic analysis, hAF-MSC and hAD-MSC were incubated with anti-CD90, anti-CD105 and anti-SSEA4 antibodies conjugated to FITC, anti-CD73, anti-HLA-DR and anti-CD34- conjugated to PercP-Cy5.5, anti-CD19, anti-CD25 and anti-CD117 conjugated to PE-Cy7, anti-CD166, CD151 and CD49c conjugated to PE (Biolegend, San Diego, CA), for 20 min followed by fixation (2% paraformaldehyde) and data acquisition in flow cytometry (BD FACS Verse, BD Biosciences).The analysis was performed using the FlowJo cytometric data analysis software.

### Production and characterization of the chitosan-xanthan scaffolds

The chitosan-xanthan scaffolds (CX) used were produced and characterized according to Westin et al. (21), using medium molecular weight chitosan with deacetylation degree around 75% (Sigma-Aldrich Co.), xanthan gum from *Xanthomonas campestris* (Sigma-Aldrich Co.), Poloxamer 188 solution at 10% (Sigma-Aldrich Co.) and acetic acid (Synth).

Briefly, 200 mL of a 1% chitosan solution (w/v) prepared in 1% (v/v) acetic acid were mixed to 215 mL of a 0.93% xanthan gum aqueous solution (w/v) at pH 7.7 containing 7% of Poloxamer 188 under controlled flow rate (10 mL/min), temperature (25 °C) and stirring (1000 rpm). The final suspension was poured into two polystyrene Petri dishes (15 cm in diameter), dried at 37 °C for 48 h and washed with deionized water to remove the Poloxamer 188 surfactant and the residual acetic acid. The scaffolds were immersed in 200 mL of 10 mM HEPES buffer for 30 min, dried at 37 °C, cutin the shape of discs of 1.5 cm in diameter, placed in 24-well plates and sterilized by exposure to ethylene oxide.

The scaffolds were characterized regarding morphology (by visual inspection, optical microscopy and SEM using a Leica microscope model LEO 440 operating at 10 kV e 50 pA), thickness (using a Mitutoyo micrometer, model MDC-25 S, Japan).

The cytotoxicity of the chitosan–xanthan gum scaffold was evaluated by an indirect extract assay by the extracts were prepared at a ratio of 0.05 g/mL in complete culture medium and incubated at 37 °C with 5% CO₂ for 1, 2, and 7 days, with daily medium replacement. Culture medium served as the negative control, whereas latex extracts were used as the positive cytotoxicity control. Cells were seeded in 96-well plates at a density of 1 × 10^6^ cells/mL and allowed to adhere for 24 h. The culture medium was then replaced with 100 µL of scaffold extracts or control media and incubated for an additional 24 h. After washing with PBS, cells were incubated with phenol red-free α-MEM containing MTT (3-(4,5-dimethylthiazol-2-yl)-2,5-diphenyltetrazolium bromide (MTT-Sigma) colorimetric assay. Scaffold (5 mg/mL) for 4 h at 37 °C. Formazan crystals were dissolved using SDS/DMSO solution containing 0.6% acetic acid, and absorbance was measured at 550 nm using a Multiskan FC microplate reader (Thermo Scientific, Finland). Cell viability was expressed relative to the negative control. The same protocol was performed for both hAF-MSCs and hAD-MSCs.

The interaction between the scaffold and hAF-MSC and hAD-MSC stromal cells was also assessed using SEM.

### Chondrogenic differentiation of human amniotic fluid and human adipose-derived mesenchymal stromal cells in chitosan-xanthan scaffold

The CX scaffolds were pre-equilibrated in the culture medium for up to 7 days prior to contacting the cells. The hAF-MSC and hAD-MSC were injected in the scaffolds with a 1 mL syringe at the concentration of 1 to 2 × 10^6^ cells in 0.5 mL of high glucose DMEM (GIBCO) culture medium supplemented with ascorbic acid (50 µg/mL, Sigma-Aldrich Co.), proline (40 µg/mL, Sigma-Aldrich Co.); insulin-transferrin-selenium (ITS + 1–1%, Sigma-Aldrich Co.), dexamethasone (0.1 µM Sigma-Aldrich Co.). Experimental groups were divided into stimulated conditions (supplemented with 10 ng/mL TGF-β3) and non-stimulated negative controls (cultured in identical differentiation medium but completely lacking TGF-β3) to properly assess growth factor-induced chondrogenesis The 24-well plates were maintained in a CO_2_ incubator at 37 °C and humid atmosphere for 7 and 21 days.

### Confirmation of chondrogenic differentiation

After the differentiation period, the samples were prepared for scanning electron microscopy (SEM) according to the protocol described by Westin et al. (Westin et al. 2017) Briefly, samples were fixed in 4% paraformaldehyde and 2% glutaraldehyde prepared in 0.2 mol/L cacodylate buffer, followed by post-fixation in 1% osmium tetroxide. Samples were then dehydrated in a graded ethanol series (50% for 15 min, 70% overnight, 90% for 15 min, and absolute ethanol three times for 15 min each), subjected to critical point drying, sputter-coated with gold, and examined using a scanning electron microscope (JEOL JSM-5800 LV).

For histological analysis, Hematoxylin-Eosin (HE), Masson’s Trichrome (MT), Picrosirius Red (PR) and Alcian Blue (AB) staining procedures were performed (Schmitz et al. 2010). Images were acquired using an optical microscope (Leica, DM2500) coupled with a digital camera and analysed using the Leica Application Suite LAS software, version 4.6.2.

Histochemical staining was semi-quantitatively assessed using ImageJ/Fiji. The entire spheroid area was defined as the region of interest (ROI; 100%), and color-based image segmentation was used to determine the percentage of area corresponding to the analyzed staining component: eosinophilic area for H&E, blue area for Alcian Blue, red area for Picrosirius Red, and blue area for Masson’s Trichrome. Representative images from each experimental condition were analyzed under the same image acquisition conditions.

For molecular analysis, we used immunohistochemistry markers for type II collagen (bs-0709R-BIOSS^®^) and aggrecan (bs-11655R-BIOSS^®^). The 5 μm cut-outs on silanized slides of the samples were treated according to the Trilogy protocol (Cell Marque^®^) followed by endogenous peroxidase blocking and overnight incubation with antibodies. We used HiDef Detection™ HRP Polymer Systromal kit (Cell Marque^®^) and DAB for detection and amplification and counter-staining with Mayer’s Hematoxylin. A slide without primary antibody was used for negative control of the reaction.

For immunofluorescence analysis, the material was deparaffinised, followed by washes with PBS 1x (pH 6,8), glycine 0.1 M, BSA block (10 g/L) and incubation with primary antibody for collagen II 1:100 (bs-0709R-BIOSS^®^) overnight in a humid chamber. Following, the samples were incubated for 1 h with Alexa 647 conjugated secondary antibody at room temperature and in a humid chamber. Finally, blocking with BSA (10 g/L) was performed, marking with DAPI, fluorescent mounting medium (DAKO) to preserve the fluorescent signal and storage at 4 °C, without exposure to light, until confocal microscope analyses (Leica, model SP8).

### Statistical analysis and experimental design

All qualitative and microstructural evaluations, including histological staining, immunohistochemistry, confocal immunofluorescence, and scanning electron microscopy (SEM), were systematically performed in independent biological triplicates (*n* = 3) per experimental group (stimulated and non-stimulated controls) to ensure the reproducibility and reliability of the findings.

## Results

### Morphological and phenotypic characterization of mesenchymal stromal cells derived from human amniotic fluid and adipose-derived tissue

**Fig. 1:**
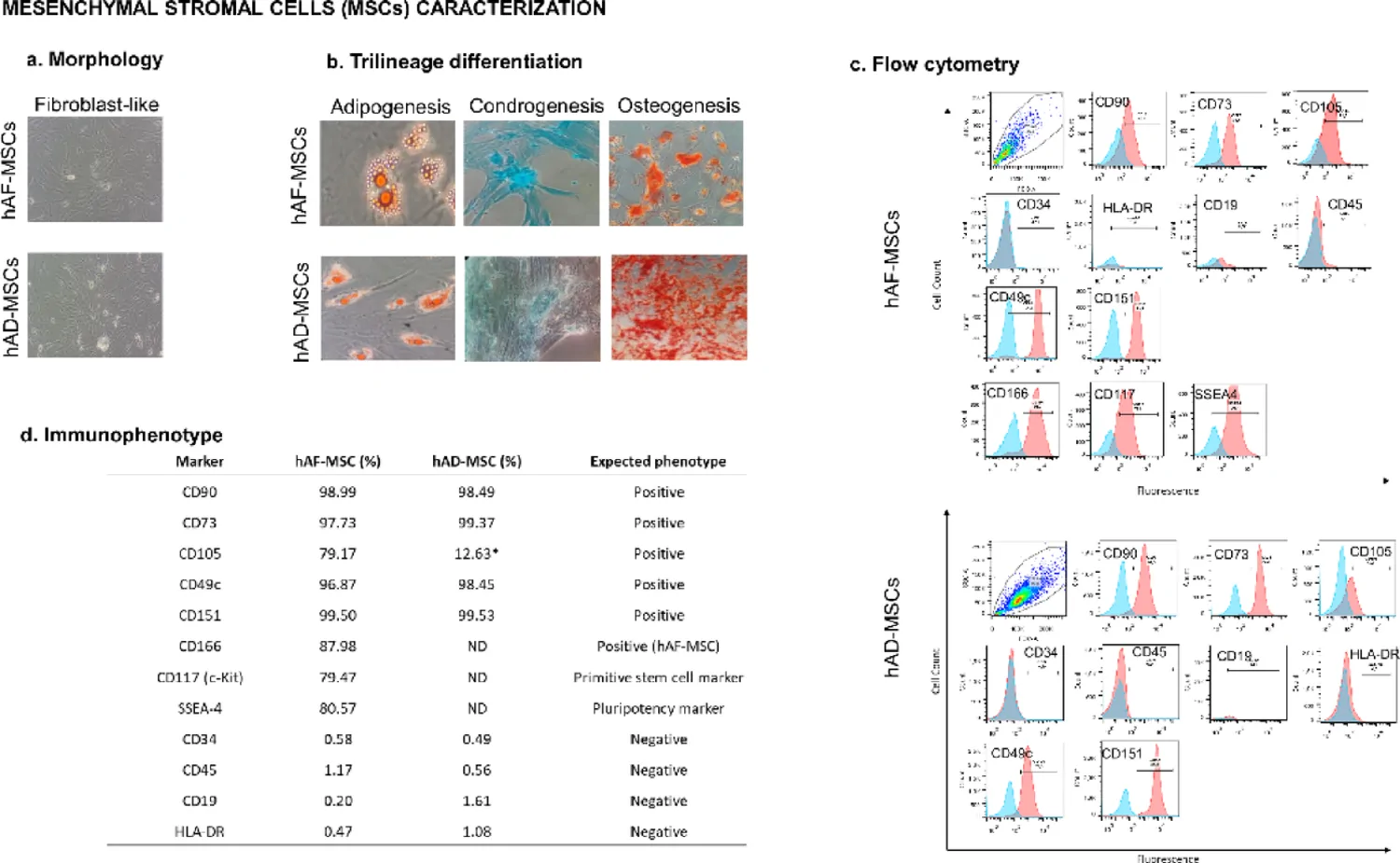
Characterization of human mesenchymal stromal cells derived from amniotic fluid (hAF-MSCs) and adipose-derived tissue (hAD-MSCs). Phase contrast microscopy of cells in culture exhibiting fibroblast-like morphology and cell adhesion (**a**). Differentiation into the three mesenchymal lineages. Adipogenic differentiation, with formation of lipid droplets stained in orange by Oil Red, chondrogenic differentiation with GAGs in blue for Alcian Blue stained; osteogenic differentiation, with calcified ECM stained in red by Alizarin Red (**b**). Analyses by flow cytometry indicating the expression of the positive markers of MSCs (CD90, CD105, CD73), negative markers of hematopoietic lineage (CD34, HLA-DR, CD19 and CD45) and positive markers chondrogenic potential (CD49c, CD151 and CD166) in both cellular sources. In hAF-MSC pluripotency markers appear positive (SSEA-4 and CD117). Immunophenotype by flow cytometry (c). Values represent the percentage of cells positive for each surface marker obtained from a representative flow cytometry analysis (**d**)

Morphological evaluation and characterization of MSCs were carried out in accordance with the recommendations established by the International Society for Cell Therapy for the characterization of mesenchymal stromal cells (Dominici et al. 2006). During their expansion phase, hAF-MSCs (Fig. 1a) and hAD-MSCs (Fig. 1d) exhibited a fibroblast-like morphology, forming characteristic structures resembling fibroblast-like colonies. No evident morphological differences were observed between hAF-MSCs and hAD-MSCs during expansion.

Both hAF-MSCs and hAD-MSCs demonstrated differentiation potential into the three expected mesenchymal lineages. Adipogenic differentiation was evident through Oil Red staining, revealing the formation of lipid droplets. Chondrogenic differentiation was confirmed via Alcian Blue staining, indicating the presence of cartilaginous ECM components. Osteogenic differentiation was detected through Alizarin Red staining, providing evidence of calcified ECM formation (Fig. 1 b).

Flow cytometric analysis confirmed the immunophenotypic profile of both cell populations prior to chondrogenic differentiation (Fig. 1c and d). Both hAF-MSCs and hAD-MSCs exhibited high expression of the mesenchymal markers CD73 (97.73% and 99.37%, respectively) and CD90 (98.99% and 98.49%, respectively). CD105 expression was high in hAF-MSCs (79.17%) but low in hAD-MSCs (12.8%). Both cell populations showed minimal expression of the hematopoietic markers CD19 (0.20% and 1.61%, respectively), CD34 (0.58% and 0.49%, respectively), CD45 (1.17% and 0.56%, respectively), and HLA-DR (0.47% and 1.08%, respectively). In addition, although both cell populations exhibited comparable expression of CD73, CD90, CD49c and CD151, hAF-MSCs differed by expressing CD117, SSEA-4 and CD166. The chondrogenic-associated adhesion markers CD49c (96.87% and 98.45%, respectively) and CD151 (99.50% and 99.53%, respectively). hAF-MSCs also expressed CD166 (87.98%), as well as the pluripotency-associated markers CD117 (79.47%) and SSEA-4 (80.57%), suggesting the retention of a more primitive immunophenotypic profile consistent with their fetal origin. Detailed flow cytometry results are summarized in Fig. 1d.

### Characterization of the chitosan-xanthan scaffold

**Fig. 2:**
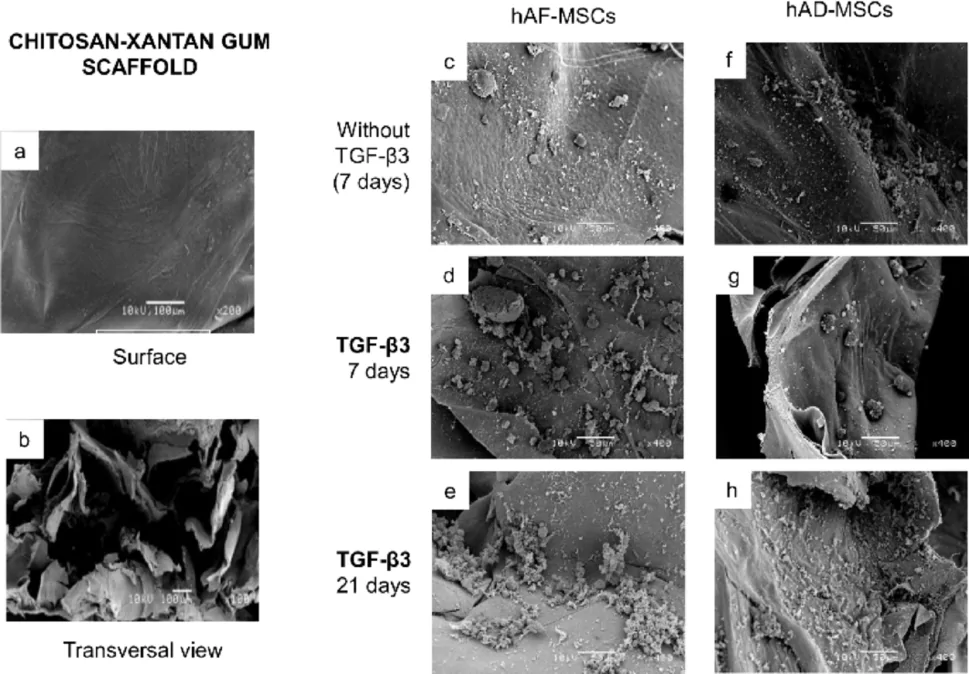
Scanning electron microscopy of chitosan-xantan gum scaffold without cells, surface (**a**) and transversal view showing interconnected pores (**b**). Chondrogenic differentiation of hAF-MSC (**c**, **d** and **e**) and hAD-MSC (**f**, **g** and **h**) in the scaffold respectively. Control group without TGF-β3 stimulation with 7 days of culture showing some attached cells on the surface of the scaffold (**c** and **f**). The group under TGF-β3 stimulation after the same period showed a greater number of adhered cells (d and g) and after 21 days, there production of fibrillar structures compatible with ECM production was observed

When wet, the scaffold had a thickness ranging from 887 to 969 μm and was flexible and opaque, exhibiting a foam-like structure. SEM analysis of the dried scaffold cross-section revealed a wavy surface (Fig. 2a) and interconnected cavities within its internal structure (Fig. 2b). Indirect MTT assays demonstrated that the scaffold extracts were non-cytotoxic to both hAF-MSCs and hAD-MSCs, in accordance with the ISO 10993-5 criterion. The complete MTT dataset, including individual measurements, mean values, standard deviations, and coefficients of variation, is provided in Supplementary Figure S1.

### Confirmation of chondrogenic differentiationin the Scaffold

After 21 days of implantation of stromal cells (SCs) into the scaffolds, macroscopic examination unveiled the presence of cell agglomerates distributed throughout the material. SEM results provided insights into the interaction between MSCs from both sources and the porous chitosan-xanthan (CX) scaffold (Fig. 2). The cells displayed robust viability, adhered effectively, proliferated, and contributed to the production of extracellular matrix (ECM) following inoculation into the scaffold.

**Fig. 3:**
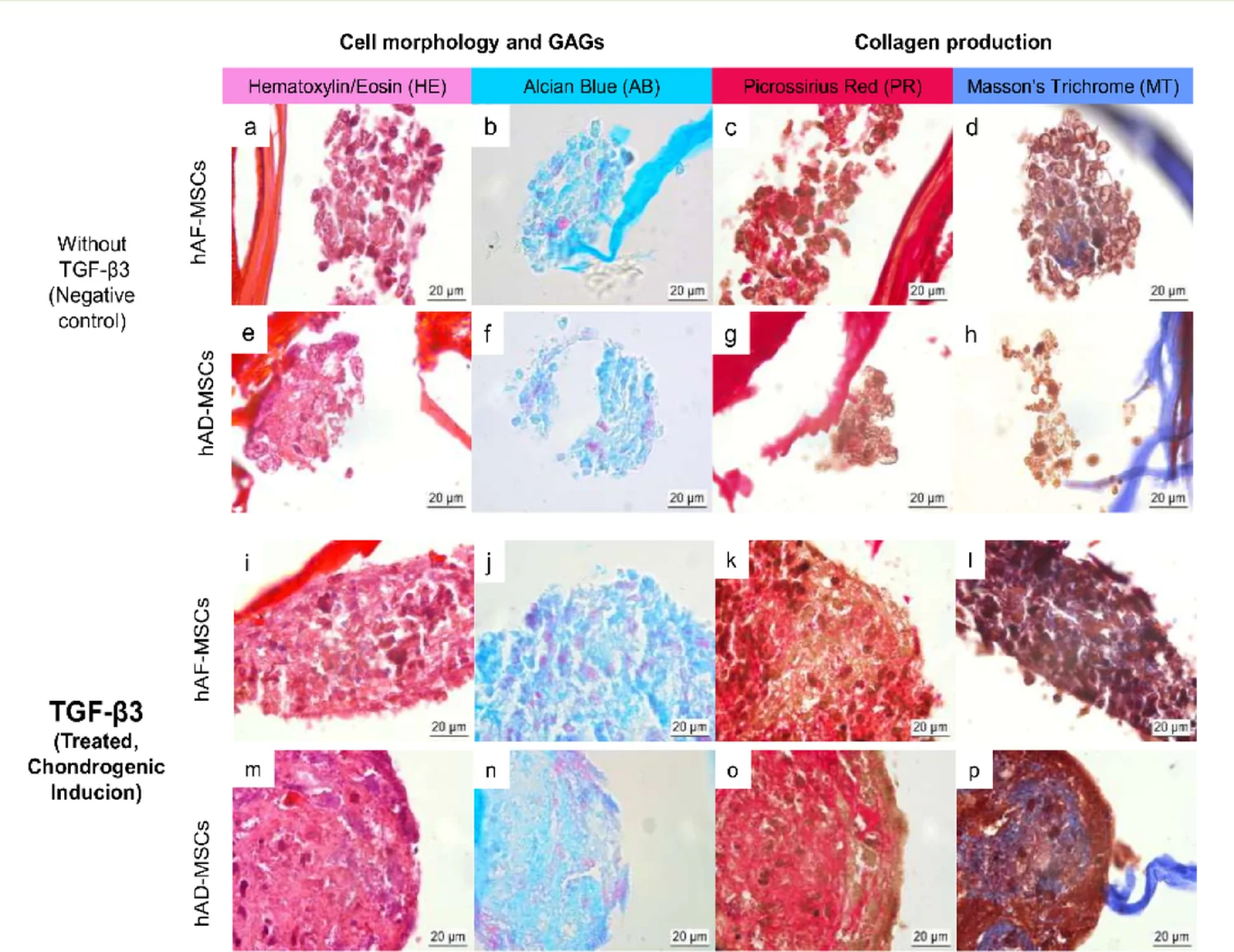
Comparative chondrogenic differentiation of hAF-MSC and hAD-MSC after 21 days into CX scaffold without and under TGF-β3 stimulation. The histological images of the group without TGF-β3 show the beginning of cellular condensation close to the scaffold and discrete ECM production (**a**–**h**). Hematoxylin and Eosin (HE) staining the intact cell nuclei stained in purple and the ECM in pink (a and e), Alcian Blue (AB) staining showing cell nuclei in pink and ECM in blue (b and f) with low ECM production, while Picrosirius Red (PR) (**c** and **g**) and Masson’s Trichrome (MT) (d and h), with specific affinity for collagen, stained the ECM in shades of pale yellow to intense red and blue, respectively. hAF-MSC (c and d) show small collagen production in red and blue, while PR and MT of hAD-MSC (g and h) show only the beginning of condensation, without ECM production. The group under TGF-β3 stimulation HE (i and m) shows a well-structured cell condensation with intense ECM production; AB (**j** and **n**) shows ECM rich in GAGs stained in blue; PR (k and o) and MT (**l** and **p**) indicate the presence of collagens

Histological analysis offered further confirmation of chondrogenic differentiation, illustrating the synthesis of collagen and glycosaminoglycans (GAGs), particularly within the group stimulated with TGF-β3 (Fig. 3). Following TGF-β3 stimulation, both hAF-MSCs and hAD-MSCs exhibited enhanced extracellular matrix deposition compared with their respective negative controls. Qualitatively, hAF-MSC constructs appeared to exhibit more intense Alcian Blue, Picrosirius Red, and Masson’s Trichrome staining than hAD-MSC constructs, suggesting greater extracellular matrix deposition under the experimental conditions.

To complement the qualitative histological assessment, semi-quantitative image analysis was performed using ImageJ/Fiji based on the representative images shown in Fig. 3. For each experimental condition, the entire spheroid area was defined as the region of interest (ROI; 100%), and color-based image segmentation was used to determine the percentage of the spheroid area corresponding to the analyzed staining component: eosinophilic area for H&E, blue area for Alcian Blue, red area for Picrosirius Red, and blue area for Masson’s Trichrome. The descriptive analysis revealed higher measured staining areas following TGF-β3 treatment for Alcian Blue, Picrosirius Red, and Masson’s Trichrome in both cell sources. Alcian Blue staining increased from 34.4% to 57.7% in hAF-MSC spheroids and from 39.5% to 65.7% in hAD-MSC spheroids. Picrosirius Red staining increased from 46.6% to 52.3% in hAF-MSC spheroids and from 39.4% to 60.3% in hAD-MSC spheroids. Similarly, the blue area measured in Masson’s Trichrome staining increased from 18.0% to 55.6% in hAF-MSC spheroids and from 7.3% to 51.8% in hAD-MSC spheroids. As the analysis was performed on representative images available for each experimental condition, the results are presented descriptively and were not subjected to statistical analysis.

**Fig. 4:**
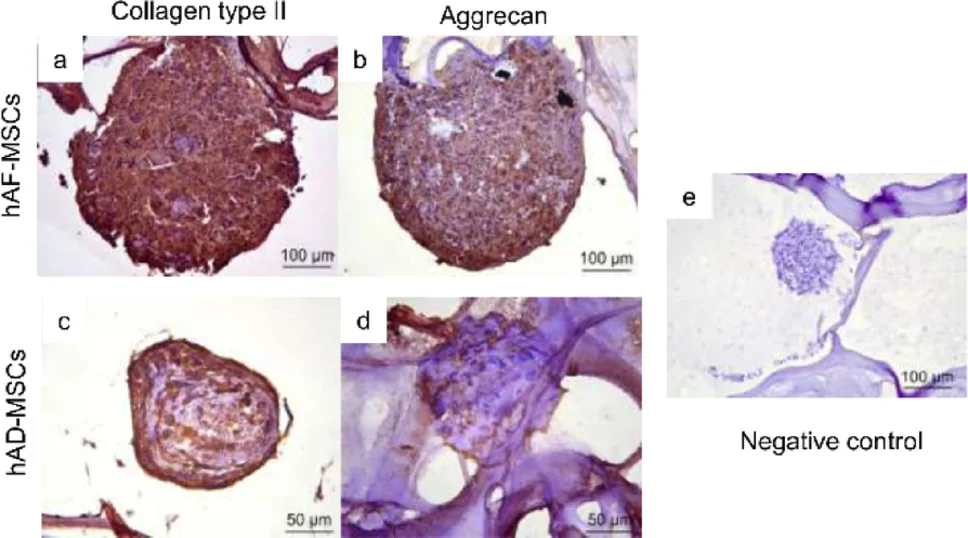
Immunohistochemistry analysis showing chondrogenic differentiation under TGF-β3 stimulation induced from hAF-MSC and hAD-MSC with positive marking for type II collagen (**a** and **c**) and aggrecan (**b** and **d**) when compared to the negative control (e)

Immunohistochemistry results affirmed the production of collagen II and aggrecan by both cell sources. Samples were counterstained with hematoxylin to mark cell nuclei in blue, and negative controls utilized samples lacking primary antibodies (Fig. 4).

**Fig. 5:**
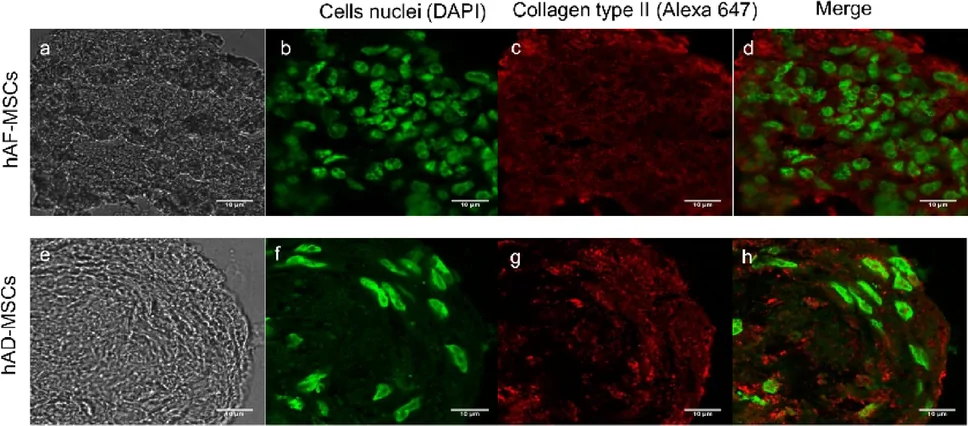
Confocal immunofluorescent analysis of hAF-MSC and hAD-MSC differentiated into chondrocytes into CX scaffold (**a** and **e**) are control images. Cells nuclei (DAPI, green) staining (**b** and **f**). Positive staining for Collagen II (bs-0709R-BIOSS^®^) conjugated with Alexa 647 secondary antibody in the ECM formed in red (**c** and **g**). Merge image (**d** and **h**)

Immunofluorescence findings exhibited positive staining for collagen II, corroborating the chondrogenic differentiation of stromal cells from both sources (Fig. 5). Intact cells, marked by cell nuclei, were observed along with a significant production of ECM.

## Discussion

The global incidence of chronic joint diseases such as OA is steadily increasing due to an aging population and rising obesity worldwide (Bijlsma et al. 2011). The inherent poor ability of articular cartilage to repair, regenerate, and remodel is one of the factors believed to facilitate the development of OA (Caldwell and Wang 2015; Onyekwelu et al. 2009; Sakata et al. 2015).There are currently no effective pharmacological treatments for OA, and in many advanced cases joint arthroplasty is the inevitable treatment for patients who have not responded to any other recommended therapies (Bannuru et al. 2019; Kolasinski et al. 2020). Consequently, there is an urgent medical need for the development of new therapies for OA such as stromal cell therapies (Diekman and Guilak 2013; Richardson et al. 2016), using different MSCs sources as well as using different sources of MSC, as well as with different biomaterials to be used as scaffolds (Zaslav et al. 2012).

The majority cell-based cartilage repair models available on the market have high costs and relatively short lifespan in terms of efficacy(Martin et al. 2019). Furthermore, to the best of our knowledge, there are no studies comparing the chondrogenic differentiation process of hAD-MSC with that of hAF-MSC. Therefore, in the present study, we assessed the potential of hAF-MSC and hAD-MSC stromal cells for chondrogenic differentiation directly into chitosan-xanthan gum scaffolds designed for cartilage tissue engineering, under TGF-β3 stimulation, analysing the suitability of a seldom used but abundant and easily obtained source of MSCs, combined with a biocompatible material easily produced, economically accessible and that does not induce inflammatory response, comparing several aspects to establish the most adequate cell source for clinical application. While hAF-MSCs are developmentally younger and embryologically closer to embryonic stem cells (ESCs), hAD-MSCs are adult mesenchymal stromal cells. In recent years, hAF-MSC has been considered a promising option to obtain MSCs, owing to the positive aspects that stimulate its application in clinical practice, such as ethical concerns, unlike the controversial use of embryonic stromal cells, hAF-MSC, but also because they are easy to obtain, easy to grow and exhibit no tumorigenicity when compared to adult stromal cells (Loukogeorgakis and De Coppi 2016, 2017).

A short time after harvesting the MSCs from both sources, the cells showed rapid adhesion and proliferation, with fibroblastic forming compatible with the characterization standards of stromal cells required by the International Society for Cell & Gene Therapy (ISCT) (Dominici, Le Blanc, Mueller, Slaper-Cortenbach, Marini, Krause, Deans, Keating, Prockop and Horwitz, 2006) (Fig. 1a and d). In the flow cytometry analysis, hematopoietic lineage markers such as CD34, CD45 and CD19 were present at levels above 2%, differently from the consensus established by Dominici(Dominici, Le Blanc, Mueller, Slaper-Cortenbach, Marini, Krause, Deans, Keating, Prockop and Horwitz, 2006). However, as observed previously by our group and by others, CD34 expression was positive in up to 50% of cells (Rossi et al. 2014; Zuliani et al. 2021). hAF-MSC contains several cell types derived from the development of the fetus and, as these cells express different markers according to their lineage and gestation time, it is believed that this peculiarity is responsible for the findings in cytometry. Although both cell populations exhibited the characteristic mesenchymal phenotype, hAF-MSCs displayed a broader expression of stemness-associated markers, including CD117, SSEA-4, and CD166, whereas hAD-MSCs showed lower CD105 expression. These findings highlight phenotypic differences between the two cell sources prior to chondrogenic differentiation.

Flow cytometry further confirmed the immunophenotypic profile of both cell populations. The exact percentages of all analyzed surface markers are presented in Table 1, providing a detailed comparison between hAF-MSCs and hAD-MSCs prior to chondrogenic differentiation.

These findings suggest that hAF-MSCs retain a more primitive immunophenotypic profile while maintaining the characteristic mesenchymal phenotype.

Considering that hAF-MSC are less mature and more variable and that this could negatively interfere with cell differentiation, immune magnetic separation was performed after the second passage of cell expansion. A subpopulation of cells positive for CD117 (Loukogeorgakis and De Coppi 2016) was selected, representing approximately 1% of the cultured cells(De Coppi, Bartsch, Siddiqui, Xu, Santos, Perin, Mostoslavsky, Serre, Snyder, Yoo, Furth, Soker and Atala, 2007). After this additional selection step, flow cytometry analysis showed high levels of SSEA-4 and other markers of chondrogenic potential (Fig. 1c and f). These findings are consistent with the enrichment of a cell population exhibiting enhanced stemness-associated characteristics while maintaining the mesenchymal phenotype.

Overall, both hAF-MSCs and hAD-MSCs exhibited comparable mesenchymal characteristics, including fibroblast-like morphology and expression of the mesenchymal markers CD73 and CD90. However, hAF-MSCs displayed a broader immunophenotypic profile, characterized by the expression of CD117, SSEA-4, and CD166, reflecting the more primitive nature of these cells and distinguishing them from hAD-MSCs prior to chondrogenic differentiation.

After the fourth passage of the SCs from both sources, it was possible to demonstrate the potential of these cells for differentiation into three mesenchymal lineages: adipogenic, chondrogenic and osteogenic, according to the pre-established consensus(Dominici, Le Blanc, Mueller, Slaper-Cortenbach, Marini, Krause, Deans, Keating, Prockop and Horwitz, 2006). The cells showed high ECM production in the differentiation process into three mesenchymal lineages. In the analysis of adipogenic differentiation, we observed the formation of lipid droplets within the newly differentiated adipocytes, in addition to ECM stained in different shades of orange by Oil Red staining. When evaluating chondrogenic differentiation, the intense production of ECM was visualised by staining with Alcian Blue, confirming the presence of proteoglycans (PGs). For osteogenesis analysis, the calcified ECM formed was stained red by Alizarin Red staining, showing the presence of calcification in significant quantities (Fig. 1b and e).

After characterization, MSCs from both sources were seeded into the scaffold. Interestingly, it was observed that, although cells from both sources differentiated into chondrocytes, cells from hAF-MSC adapted better to the scaffold than those from hAD-MSC. This lower affinity of hAD-MSC cells to the scaffold was observed by SEM, with a dispersed cell population, with fewer cells distributed over the scaffold and formation of looser fibers. Regarding TGF-β3 stimulation, in stimulated cells, both from hAF-MSC and hAD-MSC, after 21 days of culture it was possible to observe chondrogenic differentiation, with intense cell condensation and ECM production. In contrast, in groups without stimulation, there was little ECM formation (Fig. 2).

Histological analysis showed that both hAF-MSCs and hAD-MSCs underwent chondrogenic differentiation, as evidenced by cell condensation, extracellular matrix (ECM) production, and the presence of collagen and proteoglycans. The most evident difference in the histological findings was observed between TGF-β3-stimulated and non-stimulated constructs (Fig. 3). This observation was supported by semi-quantitative image analysis using ImageJ/Fiji, which showed higher measured areas of Alcian Blue, Picrosirius Red, and Masson’s Trichrome staining following TGF-β3 treatment in both cell sources. Alcian Blue area increased from 34.4% to 57.7% in hAF-MSCs and from 39.5% to 65.7% in hAD-MSCs, while Picrosirius Red area increased from 46.6% to 52.3% and from 39.4% to 60.3%, respectively. Similarly, the blue area in Masson’s Trichrome increased from 18.0% to 55.6% in hAF-MSCs and from 7.3% to 51.8% in hAD-MSCs. Although hAF-MSC and hAD-MSC constructs exhibited broadly similar patterns of differentiation, the quantitative image analysis provides an objective complementary assessment of the histological staining patterns. As this analysis was performed on representative images currently available for each experimental condition, these findings are descriptive and should not be interpreted as statistical differences.

The differentiation observed even in the absence of TGF-β3 stimulation may be related to the high concentration of cells injected together into the scaffold, potentially mimicking the cellular condensation associated with chondrogenic differentiation during embryonic development (Mello and Tuan 1999; Zuliani et al. 2018). Under these conditions, hAD-MSCs may also recapitulate a favorable cellular microenvironment. The observed increase in staining following TGF-β3 stimulation is consistent with previous studies demonstrating the influence of chondrogenic induction strategies on MSC differentiation (Glennon-Alty et al. 2013; Mara et al. 2010).

Immunohistochemical analysis confirmed the presence of type II collagen and aggrecan in constructs derived from both cell sources, further supporting chondrogenic differentiation (Fig. 4). Consistent with the histological findings, hAF-MSC constructs qualitatively exhibited greater cell condensation and more abundant extracellular matrix deposition than hAD-MSC constructs. These observations were further supported by the immunofluorescence analysis (Fig. 5).

Although the present study included semi-quantitative histological image analysis, a comprehensive quantitative assessment of chondrogenic differentiation across biological replicates and functional evaluation of the cell-scaffold constructs were not performed. The primary objective was to demonstrate the feasibility of combining hAF-MSC and hAD-MSC spheroids with the chitosan-xanthan scaffold as a cartilage tissue engineering strategy. The complementary histological, histochemical, immunohistochemical, immunofluorescence, and scanning electron microscopy analyses consistently demonstrated extracellular matrix deposition and chondrogenic differentiation. Future studies incorporating quantitative analyses of gene expression (e.g., RT-qPCR for SOX9, COL2A1, and ACAN) and protein expression (e.g., ELISA or Western blot), together with validated histological scoring, will provide additional insights into the molecular mechanisms underlying chondrogenesis and further characterize the differences between hAF-MSCs and hAD-MSCs. In addition, mechanical characterization, such as compressive modulus measurements, and quantitative degradation analysis of the CX scaffold before and after cell culture will be important to determine changes in scaffold properties during extracellular matrix deposition and to further assess its suitability for cartilage tissue engineering applications.

## Conclusions

Despite the few studies comparing human amniotic fluid stromal cells for the purpose of chondrogenic differentiation and considering the complexity of hAF-MSC due to their variability, the results of this study confirm that it is possible to differentiate in vitro stromal cells from human amniotic fluid and human adipose tissue into chondrocytes directly in the scaffold of chitosan and xanthan in the presence of TGF-β3, with evident production of an ECM rich in collagen and PGs. Taking the findings from the current study into account, we believe that future studies should assess the efficacy of this approach in chondrogenic differentiation, as well as functional results in chondral lesions in relevant preclinical and translational animal models. These efforts will help to confirm the utility of hAF-MSC for clinical use in cartilage repair and regeneration.

## Supplementary Information

Below is the link to the electronic supplementary material.

Supplementary file1 (DOCX 17492 kb)

## Data Availability

The datasets generated and analyzed during the current study are available in the Research Data Repository of UNICAMP under DOI: 10.25824/redu/LTUOZV and are available at the following URL: https://doi.org/10.25824/redu/LTUOZV.

## Abbreviations

AB: Alcian blue
BSA: Bovine serum albumin
c-Kit: Receptor tyrosine kinase involved in cell signalling and stromal cell maintenance
CX: Chitosan-xanthan gum scaffolds
DAB: 3,3′-Diaminobenzidine
DAPI: 4’,6’-Diamino-2-phenylindole
DMEM: Dulbecco’s modified eagle’s medium
ECM: Extracellular matrix
ESCs: Embryonic stromal cells
FBS: Fetal bovine serum
FIC: Free and informed consent
FITC: Fluorescein Isothiocyanate
GAGs: Glycosaminoglycans
hAD-MSCs: Human adipose-derived mesenchymal stromal cells
hAF-MSCs: Human amniotic fluid mesenchymal stromal cells
hBM-MSCs: Human bone marrow mesenchymal stromal cells
HE: Hematoxylin-Eosin
HEPES: Buffering agent used in cell culture to maintain pH stability
HLA-DR: Human Leukocyte Antigen – DR isotype
ITS: Insulin-transferrin-selenium
MSCs: Mesenchymal stromal cells
MT: Masson's trichrome
MTT: Colorimetric assay for assessing cell metabolic activity and viability
OA: Osteoarthritis
P/S: Penicillin–streptomycin
PBS: Phosphate-buffered saline
PR: Picrosirius red
SC: Stromal cells
SEM: Scanning electron microscopy
SSEA-4: Stage-specific embryonic antigen-4
TGF-β3: Transforming growth factor beta-3
WMA: World medical association
α-MEM: Alpha minimum essential medium
PGs: Proteoglycans
Scaffold: Three-dimensional structure supporting cell adhesion, proliferation, and differentiation in tissue engineering
Chondrogenic Differentiation: Process by which stromal cells develop into chondrocytes (cartilage cells).
Extracellular Matrix Production: Synthesis of proteins and polysaccharides forming tissue structure.
