# Correction: Extracellular matrix assembly during chondrogenic differentiation of amniotic fluid- and adipose-derived mesenchymal stromal cells under TGF-β3 induction

**DOI:** 10.1007/s10735-026-11022-8

**Published:** 2026-10-30

**Authors:** Carolina Coli Zuliani, Ingrid Iara Damas, Kleber Cursino de Andrade, Paulo Kharmandayan, Ângela Maria Moraes, Ali Mobasheri, Ibsen Bellini Coimbra

**Affiliations:** 1 Department of Orthopedics, Rheumatology and Traumatology, Universidade Estadual de Campinas (UNICAMP) - School of Medical Sciences, Campinas, CEP 13083-887 Brazil; 2 Department of Gynecology and Obstetrics, Universidade Estadual de Campinas (UNICAMP) - School of Medical Sciences, Campinas, CEP 13083-891 Brazil; 3 Universidade Estadual de Campinas (UNICAMP) - School of Medical Sciences, Campinas, CEP 13083-887 Brazil; 4 Department of Engineering of Materials and of Bioprocesses, Universidade Estadual de Campinas (UNICAMP) -School of Chemical Engineering, Campinas, CEP 13083-852 Brazil; 5 Department of Personalized Medicine, State Research Center for Innovative Medicine, Vilnius, LT-08406 Lithuania; 6 Health Science and Technology Research Unit, University of Oulu, Oulu, FI-90014 Finland; 7 Department of Joint Surgery, First Affiliated Hospital of Sun Yat-sen University, Guangzhou, Guangdong Province, China; 8 Faculty of Medicine, University of Liège, Liège, Belgium; 9 Department of Health Sciences, Medicine and Research, Center for Regenerative Medicine, Faculty of Health and Medicine, Krems University of Continuing Education, Krems, Austria; 10 Department of Orthopedic Surgery, Maastricht University Medical Center, Maastricht, Netherlands

**Correction to: Journal of Molecular Histology** https://doi.org/10.1007/s10735-026-10977-y

In this article the name Maria Moraes was published inadvertently.

The original article has been corrected.

