# Supplementary material for: Extracellular matrix assembly during chondrogenic differentiation of amniotic fluid- and adipose-derived mesenchymal stromal cells under TGF-β3 induction
Source: J Mol Histol. 2026 Oct 1;57(5):323. doi: 10.1007/s10735-026-10977-y (PMC13630801; doi:10.1007/s10735-026-10977-y)
Supplement: Supplementary file 1 [file 10735_2026_10977_MOESM1_ESM.docx]

MTT Cytotoxicity Assay – Chitosan-Xanthan (CX) Scaffold Extracts

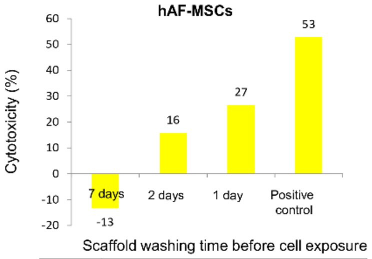

	Cytotoxicity (%)			
	7 days	2 days	1 day	Positive control
550nm			25,436	
	-13,273	15,918	30,830	54,627
	-14,225	13,379		51,137
	-12,639			
		15,918		
		18,139	23533	
**Mean**	**-13,379**	**15,838**	**26,600**	**52,882**
**SD**	**0,799**	**1,945**	**3,785**	**2,468**
**CV%**	**-5,968**	**12,282**	**14,231**	**4,667**

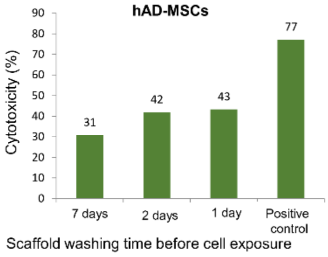

	Cytotoxicity (%)			
	7 days	2 days	1 day	Positive control
550nm	34,818	43,494	43,025	
	29,308	40,680	39,508	
	28,605	37,749	42,673	
	30,246	42,087	46,307	82,649
	27,081	43,728	45,252	78,077
	34,349	43,611	42,790	70,809
**Mean**	**30,735**	**41,891**	**43,259**	**77,179**
**SD**	**3,159**	**2,348**	**2,365**	**5,971**
**CV%**	**10,279**	**5,604**	**5,466**	**7,737**

Supplementary Figure S1. Indirect MTT cytotoxicity assay of chitosan-xanthan (CX) scaffold extracts. Cytotoxicity was evaluated using extracts obtained after different scaffold washing periods (1, 2, and 7 days) prior to cell exposure. hAF-MSCs (left) and hAD-MSCs (right) were assessed according to ISO 10993-5. Bars represent mean cytotoxicity values obtained from three independent experiments, while the accompanying tables show the individual measurements, mean values, standard deviations (SD), and coefficients of variation (CV). The positive control exhibited the expected cytotoxic response, whereas all scaffold extracts remained within the non-cytotoxic range.
